# Dr. Francis Adams

**Published:** 1861-05

**Authors:** 


DR. FRANCIS ADAMS, probably the most learned scholar in the medical profession of Great Britain, died at Banchory-Ternan, Scotland, of bronchitis, on the 26th of Feb., in his 67th year. A country surgeon, in a small village, constantly engaged in his calling, it is a matter of surprise how Dr. Adams found time to pursue his literary studies. He was best known by his masterly translation of Paulus Ægineta, which was published by the Sydenham Society, and by his translation of the genuine works of Hippocrates, which he finished in about four months. He was also the editor of Aretæus, and coeditor of "Theophilus de Fabricâ Corporis." He was peculiarly fitted for this kind of work, being an excellent Greek and Latin scholar. He also translated Hero and Leander from the Greek of Musæus; issued a work on the connection between the Greek and Latin languages; published a collection of original Latin poems, under the title of "Arundines Devæ;" wrote almost the whole of Dunbar's Greek and English Dictionary; composed various papers on "Greek Prosody;" and contributed "Notices of the Greek, Latin, and Arabic Medical Authors" for Barker's edition of Lempriere's Dictionary. Besides these, he made frequent communications on medical subjects to the British journals; his last paper being on the "Roman Bath," published in February in the *Medical Times and Gazette*.

